# Supplementary figures and images for: Rett syndrome severity estimation with the BioStamp nPoint using interactions between heart rate variability and body movement
Source: PLoS One. 2023 Mar 1;18(3):e0266351. doi: 10.1371/journal.pone.0266351 (PMC9977017; doi:10.1371/journal.pone.0266351)

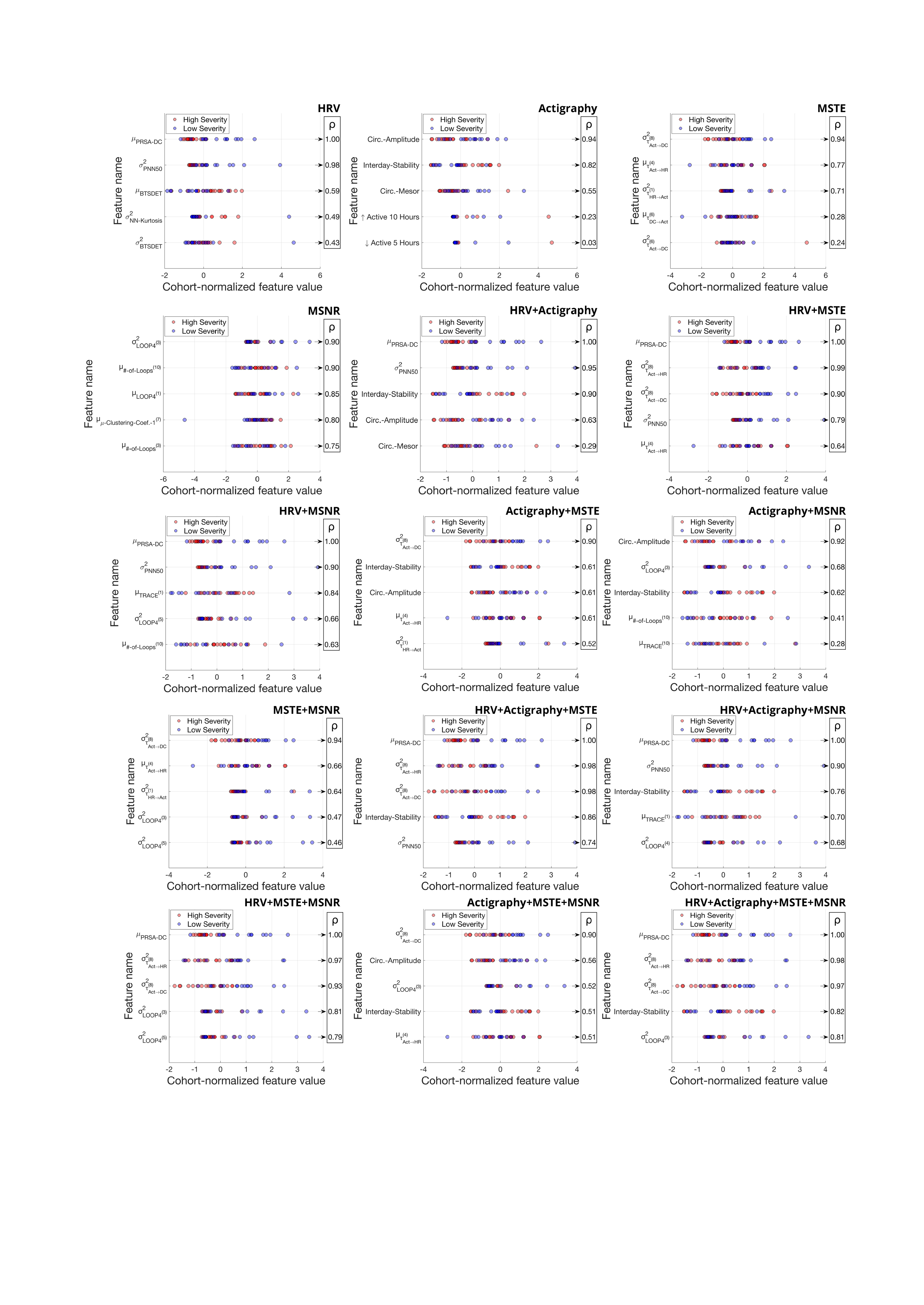

Supplement: S1 Fig — We show the normalized feature values for all 32 patient-visits for top-5 most popular features in each subplot. The data points with the red marker correspond to high-severity Rett patient-visits, and the data points with the blue marker correspond to low-severity Rett patient-visits. Further, we provide the feature popularity (ρ) scores for these five features. The individual subplots correspond to different feature combinations of the following feature sets: (1) HRV—Heart Rate Variability; (2) Actigraphy; (3) MSTE—Multiscale Transfer Entropy; (4) MSNR—Multiscale Network Representation. (TIF) [file pone.0266351.s001.tif]
